# Coronavirus Disease 2019 and Related Parkinsonism: The Clinical Evidence Thus Far

**DOI:** 10.1002/mdc3.13461

**Published:** 2022-04-28

**Authors:** Iro Boura, Kallol Ray Chaudhuri

**Affiliations:** ^1^ University of Crete, Medical School Heraklion Greece; ^2^ King's College London Department of Neurosciences, Institute of Psychiatry, Psychology & Neuroscience London UK; ^3^ Parkinson's Foundation Centre of Excellence, King's College Hospital London UK

**Keywords:** Covid, parkinsonism, Parkinson's, pathogen, pandemic

## Abstract

**Background:**

The Coronavirus disease 2019 (Covid‐19) pandemic has fueled both research and speculation, as to whether it could be a “perfect storm” for a post‐Covid emergence of parkinsonism in some susceptible individuals, analogous to the post‐encephalitic parkinsonism reported after the 1918 influenza epidemic. This theory is further augmented by reports of a pathogenic effect of the Severe Acute Respiratory Syndrome Coronavirus‐2 (SARS‐CoV‐2) on the central nervous system with specific impact on the dopaminergic pathway, as well as the possibility of the virus to selectively bind to Angiotensin‐Converting Enzyme‐2 (ACE‐2); these molecules are expressed abundantly in the midbrain dopamine neurons and, are likely involved in several cellular mechanisms cited in Parkinson's Disease (PD) pathophysiology.

**Objectives—Methods:**

Therefore, we performed a review of the literature up to February 2022 to explore the current landscape considering published cases of new‐onset parkinsonism after a SARS‐CoV‐2 infection in otherwise healthy individuals. We summarized their clinical features, diagnostic and treatment approaches, discussing potential underlying mechanisms in light of PD pathogenesis theories.

**Results:**

Twenty cases that developed parkinsonian features simultaneously or shortly after a reported SARS‐CoV‐2 infection were reviewed. In 11 of them, parkinsonism appeared in the context of encephalopathy, while four patients developed post‐infectious parkinsonism without encephalopathy, and four bore similarities to idiopathic PD. Nine patients exhibited a good response to dopaminergic therapy, while four responded to immunomodulatory treatment.

**Conclusions:**

Available data does not yet justify a clear association between the Covid‐19 pandemic and a parkinsonism wave. However, vigilance is necessary, as long‐term effects might have not been revealed.

The outbreak of Severe Acute Respiratory Syndrome Coronavirus‐2 (SARS‐CoV‐2) has brought into surface older theories about the role of infectious agents in the pathogenesis of Parkinson's Disease (PD).^1^, ^2^ The idea of an upcoming parkinsonism wave due to the worldwide spread of SARS‐CoV‐2, with Coronavirus disease 2019 (Covid‐19) unmasking future cases of parkinsonism, has been recently much discussed and constitutes a case of great concern to many, particularly for those who may have developed severe persistent anosmia or hyposmia following Covid‐19.^3^, ^4^, ^5^ These speculations have been underpinned by historical documentation of parkinsonism cases reported in the acute or chronic phase of encephalitis lethargica (EL), an entity which affected more than 1 million people in Europe, North and Central America and India from 1916 to 1930.^6^ Although the etiological substrate of EL remains largely a mystery, many consider an epidemiological link to the Spanish influenza pandemic, which is thought to have killed more than 40 million people worldwide in 1918–1919 and whose strains disappeared in 1933, thus coinciding with the EL period.^6^ Indeed, it has been found that those born between 1888 and 1924 had a 2‐ to 3‐fold risk of developing PD compared to those born outside of this time period.^7^

The association of numerous viruses with the development of persistent or transient parkinsonism has also been well‐documented (Table 1). A “deep dive” into the so far published cases of parkinsonism following Covid‐19 can therefore help unravel whether a viral etiology is possible, or the association is merely coincidental.

**TABLE 1:** *Viruses connected with parkinsonism cases or dopaminergic neurons pathology* ^8^, ^9^, ^10^, ^11^, ^12^, ^13^, ^14^, ^15^, ^16^, ^17^, ^18^, ^19^, ^20^

Pathogens	Para‐ or Post‐infectious parkinsonism	↑risk for PD	Animal models	Cell lines
Coxsackie virus	+^a^			
Dengue virus	+^a^			
Epstein Barr virus (EBV)	+^a^			
Influenza A, H1N1			+	+
Hepatitis C virus (HCV)		+		
Hepatitis E virus (HEV)	+^a^			
Human Immunodeficiency virus (HIV)	+			
Japanese encephalitis B virus (JEBV)	+^a^		+	
Measles virus	+^a^			
St Louis encephalitis virus	+^a^			
Theiler's murine encephalomyelitis virus (TMEV)			+	
Western equine encephalitis virus (WEEV)			+	
West Nile virus (WNV)	+^a^		+	

^a^ related to encephalitis.

PD, Parkinson's disease.

We review here a series of published cases that developed parkinsonian signs concurrently or shortly after a confirmed SARS‐CoV‐2 infection.

## Methods

We conducted a descriptive review of papers in English indexed in PubMed/ MEDLINE database from January 2019 to February 2022, reporting patients who developed parkinsonism at the onset or after a reported SARS‐CoV‐2 infection. We used the following search terms, either as plain text or MeSH terms: “Parkinson's Disease”, “parkinsonism”, “parkinson”, “extrapyramidal”, “tremor”, “Covid 19”, “Covid”, “SARS‐CoV‐2”, “coronavirus.” Cases of patients presenting solely with tremor or combined with other hyperkinetic movement disorders without evidence of concomitant bradykinesia or rigidity, as well as cases of parkinsonism appearing after Covid‐19 vaccination, were excluded. The severity of SARS‐CoV‐2 infection on each occasion was determined according to globally accepted guidelines of the National Institute of Health.^21^

## Results

**FIG. 1:**
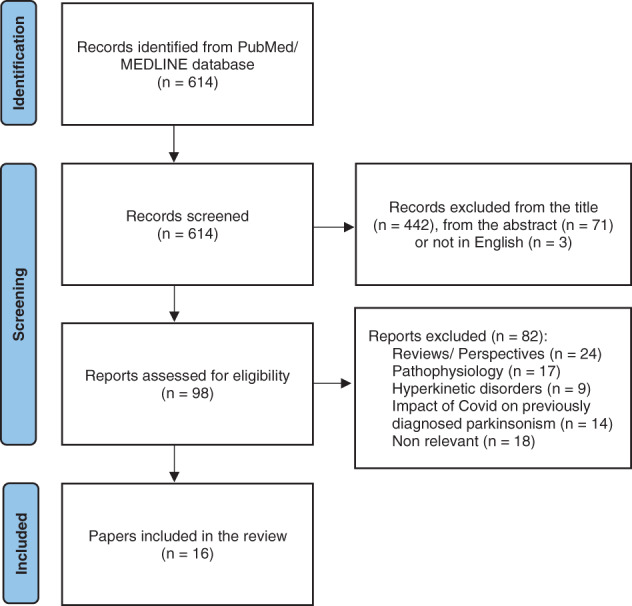
Prisma flowchart for a review of articles on “Coronavirus Disease 2019 and related Parkinsonism: the clinical evidence thus far” from PubMed database on February 14, 2022.

From the searching procedure, and after removing duplications, we screened 614 records. 442 were excluded from title and an additional 71 from the abstract, as being irrelevant to our scope. Three items were not written in English and were also withdrawn. We evaluated 98 full‐text papers for eligibility and excluded 82 due to various reasons (Fig. 1).

A total of 16 papers were included, describing 20 cases that met our criteria (Table 2).^22^, ^23^, ^24^, ^25^, ^26^, ^27^, ^28^, ^29^, ^30^, ^31^, ^32^, ^33^, ^34^, ^35^, ^36^, ^37^ In 13 patients, SARS‐CoV‐2 infection was confirmed with a nasopharyngeal swab, found positive for SARS‐CoV‐2 in a real time reverse‐transcription polymerase chain reaction (RT‐PCR) assay. For the residual seven cases reported to have been diagnosed with Covid‐19 (patients 7, 8, 10, 11, 15, 16, 20) the diagnostic means of confirmation were not provided. Covid‐19 was reported of mild severity in four patients, of moderate severity in four patients, and of severe in 11, while no information was provided for patient 4. The majority of patients had no history of prodromal PD symptoms (90.9%), such as constipation, rapid eye movement (REM) sleep behavior disorder (RBD) or hyposmia, but such symptoms were not investigated in nine of them. Family history of PD or tremor was only checked in four patients and was negative in all of them. A prior use of neuroleptic drugs was mentioned in four out of 11 patients (36.3%).

**TABLE 2:** Characteristics of parkinsonism cases in the context of Covid‐19

ID	Age/Sex	Onset	Covid‐19 severity	Signs of encephalopathy	Asymmetrical parkinsonism	Duration	ICU	Response to Immuno‐ modulatory treatment	Response to dopaminergic drugs	Follow‐up	Prodromal PD	Family history	Parkinsonism‐inducing drugs history	CSF	MRI	DaTscan
#1(9)	35F	10d	Mild	−	+	Persistent	−	−	Significant			−	−	normal	unremarkable	abnormal
#2(10)	58 M	38d^a^	Severe	+	+	Persistent	+	−	None	53d	−	−	−	normal	unremarkable	abnormal
#3(11)	45 M	14‐21d	Moderate	−	+	Persistent	−	None	Significant		−	−	−	normal	unremarkable	abnormal
#4(12)	73 M	0		+		Persistent									abnormal	
#5(13)	72 M	2d	Severe	+	−	Transient	+^b^	Good		2mo			−		unremarkable	
#6(14)	64F	5d	Mild	−	+	Persistent	−	−			+					abnormal
#7(15)	60 M	8d^a^	Severe	+	N/A	Transient	+		Significant	4mo			−		abnormal	
#8(16)	46 M	38d^a^	Severe	+	+	Persistent	+	−	None	1y					abnormal	
#9(17)	40F	22d^a^	Severe	+	+	Transient	+	Good		143d			+	normal	unremarkable	
#10(18)		3mo	Severe				+									
#11(19)	65F	6d	Moderate	+	−	Persistent	−	−	Significant	4mo		−	−	normal	abnormal	
#12(20)	35F	7‐14d^a^	Severe	+	+	Persistent	+		Significant	Not specified	−		−	normal	abnormal	
#13(21)	70F	31d	Severe	+	+	Persistent	−	Good	Modest	9mo	−		+	normal	unremarkable	abnormal
#14 (21)	73F	0	Mild	+	−	N/A^c^		None	None	30d^c^	−		+	abnormal	unremarkable	
#15(22)	67 M	4mo	Moderate	−	+	Persistent	−	−			−				unremarkable	abnormal
#16(22)	45 M	3mo	Mild	−	+	Persistent	−	−			−		−		unremarkable	abnormal
#17(23)	31 M	6d	Severe	+	−	Transient	−	Good					+	abnormal	abnormal	
#18(24)	72 M	14d	Severe	−		Persistent			Significant	4mo	−		−			
#19(24)	66 M	14d^a^	Severe	−	+	Persistent	+		Significant	1mo	−		−	normal	unremarkable	
#20(24)	74 M	21d	Moderate	−		Persistent			Significant	6mo	−		−	normal	unremarkable	

^a^ Onset within or after admission to ICU, whether intubated or not.

^b^ The patient was admitted in the ICU after the transient episode of parkinsonism.

^c^ Death.

CSF, cerebrospinal fluid; Covid‐19, Coronavirus Disease 2019; d, day; DaTscan, Dopamine Transporter scan; F, female; ICU, Intensive Care Unit; ID, identification; M, male; mo, months; MRI, magnetic resonance imaging; N/A, not applicable; PD, Parkinson's disease; y: year.

The onset of parkinsonism, which was asymmetrical in 11 out of 15 cases (73.3%), occurred within one week after the Covid‐19 diagnosis in six patients, within one month in eight patients, and up to four months in six patients. Although patient 7 manifested asymmetrical bradykinesia of the right limbs, his left side could not be properly assessed due to hemiparesis after a stroke. On six occasions parkinsonism was noted within or after the discharge of the patient from the Intensive Care Unit (ICU), when level of consciousness had improved. Patient 5 had presented with bradykinesia before being admitted in the ICU, while no information was provided on the temporal relationship of parkinsonism detection and ICU admission for patient 10. Signs of encephalopathy were found in 11 cases out of 19 patients (57.9%), occasionally including alterations in consciousness, ocular abnormalities, myoclonus, and epileptic phenomena (Table 3). Hyposmia or anosmia in the setting of Covid‐19 was reported in the nine out of 11 patients (81.8%).

**TABLE 3:** Co‐existent neurologic manifestations along with parkinsonism

ID	Ocular abnormalities	Consciousness impairment	Hyperkinetic movement disorders	Pyramidal signs	Epileptic phenomena	Behavior impairment	NMS
#1(9)							Hyposmia
#2(10)	+^a^	+	Myoclonus, tremor	+			Hyposmia
#3(11)			Tremor				Hyposmia, frequent urination
#4(12)		+					
#5(13)		+	Tremor				
#6(14)			Tremor				Hyposmia, constipation, fatigue
#7(15)		+		+			
#8(16)							
#9(17)			Tremor, stereotypical/choreiform movements		+	+	Hyposmia
#10(18)							
#11(19)		+					Nausea
#12(20)		+	Tremor			+	Hyposmia, drooling, hallucinations
#13(21)	+	+	Myoclonus, dystonia		+	+	Cognitive impairment
#14(21)	+	+	Tremor		+^b^		
#15(22)			Tremor				Hyposmia
#16(22)			Tremor				Hyposmia
#17(23)		+	Myorhythmia			+	
#18(24)							Hyposmia, orthostatic hypotension
#19(24)					+^c^		
#20(24)							

^a^ including opsoclonus.

^b^ only epileptiform discharges in EEG.

^c^ prior history of epileptic seizures.

ID, identification number; NMS, non‐motor symptoms.

With three exceptions, brain magnetic resonance imaging (MRI) was performed in all cases and was found abnormal in six (35.3%). More specifically, an ischemic stroke was found in the basal ganglia and corona radiata of patient 7. In patient 8, there was oedema of the globus pallidus and microbleeds in cerebellar nuclei, attributed to hypoxia; these lesions evolved into atrophy of the respective loci in subsequent imaging. Symmetrical and non‐enhancing lesions with an increased T2 signal in the pallidum were found in the MRI of patient 12, with the authors also mentioning the possibility of silent hypoxia. Patient 11 demonstrated symmetrical T2 hyperintensities in the area of the caudate and putamen, sparing the globus pallidus; these lesions exhibited diffusion restriction and were attributed to extra‐pontine osmotic demyelination due to subjacent uncontrolled hyperglycemia. An increased T2 signal was found in both thalami of patient 17 with concurrent hemosiderin deposition and patchy contrast enhancement. A subtle T2 hyperintensity was also found in the pons of this patient, leading the authors to a diagnosis of acute necrotizing encephalopathy (ANEC). Finally, an increased signal of the frontal lobes in T2 sequences was reported in the brain MRI of patient 4 without any further clarifications given.

Seven patients underwent dopaminergic uptake imaging (6‐[18F]‐L‐fluoro‐L‐3,4‐dihydroxyphenylalanine (F‐FDOPA)‐based positron emission tomography (PET) for patient 3, dopamine transporter single‐photon emission computerized tomography (SPECT) imaging with ioflupane I‐123 injection (DaTscan) for the rest). All of them exhibited decreased dopamine uptake either in one or both putamina similarly to typical PD. However, the authors of one case (patient 1) commented that the dopaminergic deficit was more pronounced in the mid‐putamen, and not the posterior part, differentiating this pattern from what would be classically expected in PD. In five patients (patients 1, 2, 3, 6, 13) the depicted deficit in the nigrostriatal pathway complied with the side clinically more affected by parkinsonism. In the other two cases (patients 15, 16) the deficit was bilateral, although one side of the body was clinically more severely affected. Four patients underwent a brain FDG (2‐deoxy‐2‐[18F]fluoro‐D‐glucose)‐based PET scan. Patients 13 and 14 demonstrated a diffuse cortical hypo‐metabolism, bearing similarities to some autoimmune encephalitis cases, along with increased glucose metabolism in the mesial temporal lobes, basal ganglia, and brainstem.^38^, ^39^ Patient 9 showed increased glucose metabolism in the mesial temporal lobes and subthalamic nuclei, which normalized after the administration of intravenous immunoglobulin therapy (IVIg). The examination was normal for patient 1.

A lumbar puncture was performed on 11 cases, with only patients 14 and 17 showing signs of inflammation in the cerebrospinal fluid (CSF). In patient 9, IgG antibodies for SARS‐CoV‐2 were found in the CSF along with elevated proinflammatory cytokines. Seven patients (patients 2,3,9,11,13,14,17) underwent a screening for a range of serum and/ or CSF antibodies usually associated with autoimmune encephalitis with negative results on all occasions.

Genetic testing was performed on three cases with patients 15 and 16 testing positive for a heterozygous mutation in the genes of glucocerebrosidase (GBA) and leukine‐rich repeat kinase 2 (LRRK2) respectively. A genetic substrate was not confirmed for patient 3.

Immunomodulatory/ immunosuppressive treatment was administered in six cases during the acute phase with no effect on two of them. Dopaminergic treatment with levodopa or dopamine agonists was administered to 12 patients with no effect on three of them. Parkinsonism was transient on four occasions. Patient 5 exhibited a full recovery, 24 hours after convalescent plasma therapy administration. Patient 7 experienced a full resolution of symptoms after receiving a regimen with levodopa/ carbidopa and modafinil for one month. Parkinsonism had not re‐appeared after a one‐month follow‐up assessment. Patients 9 and 17 also demonstrated full recovery following treatment with two cycles of IVIg and high doses of methylprednisolone, respectively.

Duration of follow‐up varied greatly, ranging from 1 month to 1 year; however, it was missing in almost half of the cases. One death was reported 30 days after the patient's discharge from hospital, resulting from the sequelae of the patient being bedridden and incontinent (patient 14).

## Discussion

We reported 20 published cases of parkinsonism, occurring concurrently or shortly after a SARS‐CoV‐2 infection from a detailed perusal and review of literature since the beginning of the pandemic. In 11 (55%) of these cases (patients 2, 4, 5, 7–9, 11–14, 17), parkinsonism developed in the setting of encephalopathy. Four patients (patients 1, 18–20) demonstrated parkinsonism without encephalopathy with the authors placing them in the spectrum of post‐infectious parkinsonism, while four individuals (patients 3, 6, 15, 16) were diagnosed with parkinsonism in the setting of probable PD. No clarifications were provided for patient 10.

Secondary persistent or transient parkinsonism due to viruses constitutes a well‐described entity, presenting either acutely or sub‐acutely (Table 1), similarly to our case series. The temporal proximity of the emerging parkinsonian features with a Covid‐19 diagnosis, along with the co‐existent encephalopathy in some of the above patients, led the authors to assume an etiological connection between the two. We would think some of these cases may well fit the concept of general viral post‐encephalitic parkinsonism, as has been described in the past, following a range of viral encephalopathies (Table 1). Interestingly, apart from parkinsonism, the clinical picture of EL included ocular and consciousness level abnormalities with somnolence, along with other movement disorders,^6^ bearing similarities to patients 2, 13 and 14. The frequency of encephalopathy in the context of Covid‐19 seems to vary widely (7–69%),^40^ while in a large group of 129,008 Covid‐19 patients of all ages, 138 cases of encephalitis have been confirmed, generating an incidence of 0.215%.^41^

The clinical symptomatic aspect of the neuroinvasion potential of SARS‐CoV‐2 has been cited on many occasions with headache, dizziness and hyposmia being the most regularly reported Covid‐19 manifestations.^42^ A few molecules/ receptors have been speculated to facilitate the SARS‐CoV‐2 entry and spread in the CNS, including ACE2, Transmembrane Serine Protease 2 (TMPRSS2) and Neuropilin 1 (NRP1), with the former being the most, allegedly, common target.^43^ ACE2 is highly expressed in the alveolar epithelial type II cells, but is also found in numerous extrapulmonary tissues, such as the vascular and the intestinal endothelial cells and the brain,^44^ including the striatum.^45^ Using next generation sequencing techniques, ACE2 has been recently detected in the substantia nigra and the olfactory bulb,^46^ while autopsy studies have also confirmed a high expression of NRP1 in the olfactory epithelium.^47^

The idea that SARS‐CoV‐2 enters the CNS from the periphery through either the olfactory or the vagus nerve has gained popularity, as anterograde promotion of viral vectors to more rostral CNS sites has been confirmed in the past using animal models.^48^ The olfactory bulb is not protected by the blood–brain‐barrier (BBB), constituting, theoretically, an easier target for airborne viruses. The SARS‐CoV‐2 viral load, assessed using RT‐PCR and in situ hybridization and immunohistochemical staining techniques, has been found increased in the nasal epithelium.^49^ Moreover, a recently published study, assessing brain changes in a sample of 785 UK Biobank participants (401 Covid‐19‐positive individuals and 384 controls), who had undergone a brain scan before and after being infected with SARS‐CoV‐2, has revealed a significant reduction in gray matter thickness and tissue‐contrast in the orbitofrontal cortex and parahippocampal gyrus, along with significant alterations in brain regions functionally linked to the primary olfactory cortex, suggestive of tissue damage.^50^ Since hyposmia or anosmia are prominent symptoms in Covid‐19,^42^ Douaud and colleagues have noted that such brain changes might further support a spread of degeneration or inflammation through the olfactory pathway, although the persistence of these phenomena needs to be further explored. Indeed, nine out of 11 patients in our case series, whose olfaction was examined, reported hyposmia. The vagus nerve is anatomically connected both to the respiratory and the gastrointestinal system. Past experiments in mice have confirmed the transvagal transmission of Influenza A virus from the respiratory mucosa to the basal ganglia,^51^ while there is data supporting the potential of SARS‐CoV‐2 entry through the intestine.^52^ According to the dual‐hit hypothesis of PD pathogenesis, a neurotropic pathogen, like a virus, has been speculated to enter the CNS through the nasal or gastric pathway, both of which appear to constitute sites of early pathology in PD.^1^, ^53^

SARS‐CoV‐2 has the potential to infect immune cells as well, causing a cytokine release and triggering an excessive immune response in the periphery.^46^ Such mechanisms can affect the BBB permeability, allowing infected immune cells or the virus per se to invade the CNS and induce vascular damage. An autopsy study of 43 Covid‐19 patients has shown activation of microglia and CNS infiltration by cytotoxic T‐lymphocytes, more apparent in the brainstem.^54^ This population of immune cells might induce a secondary cytokine storm inside the brain.^44^ Interestingly, increased proinflammatory cytokines and a high titer of anti‐SARS‐CoV‐2 IgG antibodies were detected in the CSF of patient 9, which is a previously described, although rare phenomenon.^55^ Whether these antibodies were produced locally or crossed the BBB due to the systemic inflammation remains unclear. Neuroinflammation has been considered a substantial contributor in PD pathogenesis,^56^ promoting neurodegeneration, with midbrain dopamine neurons being particularly vulnerable to systemic inflammation due to high energy requirements.^57^ An autoimmune mechanism was also suspected to have mediated EL in the past.^58^ Corticosteroids, IVIg, plasmapheresis or monoclonal antibodies administration have been included in the Covid‐19 treating protocols across different clinical settings with occasionally satisfactory results, suggesting an immune‐mediated substrate of the symptoms, although, similarly to our case series, the response was not constant on all occasions.^59^, ^60^ Interestingly, the brain MRI of patient 17 suggested a diagnosis of ANEC, a rare, but distinctive type of usually virus‐related acute encephalopathy, which has been associated with better outcomes when steroids are administered early,^61^ like in this particular case.

Αll seven patients who underwent functional nigrostriatal imaging showed a decreased dopamine uptake in the striatum, revealing a deficit in the dopaminergic nigrostriatal pathway, which, although suggestive of a parkinsonian syndrome, is not diagnostic of PD. Although such findings in nuclear imaging usually refer to an older, possibly ongoing, brain lesion,^35^ there have been reports of decreased nigrostriatal uptake developing acutely or sub‐acutely.^62^, ^63^ These latter lesions are usually accompanied by abnormal findings in the brain MRI scan as well, which was not the case in these seven patients.

With a wide range of investigations available nowadays, other acute causes of secondary parkinsonism should also be excluded. Parkinsonism in patient 11, who had a history of diabetes mellitus, was initially thought to be metabolic due to the subjacent uncontrolled hyperglycemic state (diabetic striatopathy), which was triggered after dexamethasone administration according to Covid‐19 therapeutic protocols. However, the persistent nature of the symptoms after hyperglycemia correction, and the brain MRI findings led to a diagnosis of extra‐pontine osmotic demyelination, which has been associated with de novo movement disorders due to disruption of the striato‐thalamo‐cortical network.^64^ Authors suggested that a potential disruption of the BBB due to the Covid‐19‐precipitated inflammation could have also contributed.

After reviewing the imaging findings of patient 8 and 12, authors attributed their symptoms to potential hypoxic – ischemic changes of the brain due to disturbance of the respiratory system and hypoperfusion, suggesting that respiratory abnormalities, typically accompanying SARS‐CoV‐2 infection, might play a crucial role in the development of neurological manifestations. Interestingly, most cases associated with encephalopathy in our case series were related to a severe SARS‐CoV‐2 infection and, thus, a greater compromise of the respiratory system. However, parkinsonism, but also other neurological symptoms, have been reported in the absence of respiratory symptoms,^40^ suggesting a more direct CNS insult of the virus.

Vascular damage constitutes a recognized complication of Covid‐19.^65^ According to Brundin and colleagues, after SARS‐CoV‐2 gains access to the bloodstream, it can invade endothelial vasculature cells, which in combination with the hypercoagulable state induced by Covid‐19 might affect the nigrostriatal pathway similarly to vascular parkinsonism.^5^ In our case series, patient 7 presented with left‐side hemiparesis and right‐side bradykinesia, while the brain MRI revealed an ischemic stroke of the basal ganglia and the corona radiata (without the authors specifying the side of the lesion or providing images).

Considering the four cases bearing similarities to PD, they all exhibited asymmetrical parkinsonism and decreased dopaminergic uptake in functional nigrostriatal neuroimaging with unremarkable findings in the brain MRI scan and lumbar puncture, when performed. Patient 3 exhibited an excellent response to levodopa, while there was no mention of therapeutic strategies for patients 6, 15 and 16. According to the authors, all four cases could be clinically considered as cases of probable PD, although the possibility of post‐infectious parkinsonism could not be excluded. The pathogenesis of PD, along with other parkinsonism‐related neurodegenerative disorders, remains largely a mystery, with environmental factors assumed to play a leading role, especially after the age of 50.^66^ In this context, the role of pathogens remains highly disputable and the contribution of a combination of environmental factors with or without a susceptible genetic substrate cannot be excluded.^3^ Three of the above cases underwent a genetic testing and a subjacent mutation was found in two of them (patients 15, 16), leading the authors to assume that SARS‐CoV‐2 might have triggered a transition of prodromal to symptomatic PD.

According to the multiple‐hit hypothesis, a viral‐based inflammation is one of the potential factors rendering the CNS susceptible to preceding or subsequent insults from accumulating stressors, serving as a trigger in the emergence of parkinsonism.^2^ It was recently demonstrated in a meta‐analysis of retrospectively collected data that people with a past history of a confirmed infection (bacterial, viral, mycobacteria, or generic CNS infection) bore a 20% higher risk for developing PD in the future compared to controls.^67^ However, the group of studies examined was considerably heterogeneous, while the subgroup analysis showed a statistically significant association only for bacterial, and not viral infections. A Swedish group of researchers also described an association between past CNS infections and the subsequent manifestation of PD.^68^ This association was found even stronger in individuals with multiple hospitalizations due to CNS infections. The viral hypothesis was further supported by epidemiological data revealing a higher frequency of PD among occupations with an increased risk for respiratory infections, like teachers and healthcare workers (“clustering of PD” theory).^69^

Different mechanisms underlying those cases of supposedly para‐ or post‐infectious parkinsonism have been described in the literature, including structural or functional impairment of the nigrostriatal pathway, inflammatory or vascular damage in cases of co‐existent encephalopathy or unmasking of already active, though asymptomatic, prodromal PD.^70^ We believe that our case series is roughly in accordance with the above mechanisms (Fig. 1).

Our review of these 20 newly‐onset parkinsonism cases and any potential links to Covid‐19 is not without limitations. The design of our review and the number of cases reported do not allow us to assume any causality between Covid‐19 and the emerging parkinsonian symptoms, as the possibility of chance or of exterior factors cannot be overlooked. The underrepresentation of mild Covid‐19 cases may lead to bias in the reported prevalence rates, while we cannot ignore that the popularity of the topic about a potential post‐Covid parkinsonism wave might have led to publication bias. A key issue is that none of our cases had their neurological status registered before being diagnosed with SARS‐CoV‐2; indeed, in a recent editorial it was commented that mild parkinsonian symptoms might have pre‐existed in some patients and, hence, the relationship with Covid‐19 may be considered tenuous.^71^ Moreover, many of the reported cases had only short follow‐up periods or no follow‐up at all. Although more than half of the patients in our case series underwent a lumbar puncture, an acute SARS‐CoV‐2 CNS infection was not confirmed in any of them. This is consistent with the majority of SARS‐CoV‐2 patients investigated for other types of neurological symptoms, who, not only tested negative for SARS‐CoV‐2 in CSF RT‐PCR, but also had relatively unremarkable findings in the CSF analysis.^72^ It is also of interest that four patients in our case series had a history of neuroleptic drug use preceding the emergence of parkinsonism (chronic use for patient 14 due to anxiety‐depressive disorder with psychotic features, temporary use for patients 9, 13 and 17 due to agitation and hyperkinetic movements respectively in the context of SARS‐CoV‐2). Although the abnormal result of nigrostriatal neuroimaging might exclude the possibility of acute drug‐induced parkinsonism in patient 13, this possibility cannot be overlooked for the other patients.

With more than 5300 confirmed cases of Covid‐19 per 100,000 globally (as of February 2022)^73^ and an annual incidence of about 15 PD cases per 100,000,^74^ anticipating a parkinsonism wave based solely on the current 20 published cases may be premature. A thorough neurological examination, including screening for parkinsonism, is not common in general practice, although such a process is required if we are to address a potential emergence of post‐Covid parkinsonism. Indeed in the United Kingdom, the National Health System (NHS England) commissioned a number of universities to organize the Covid‐CNS study, aiming to address such investigations in Covid‐19 patients, including brain imaging in infected individuals with serial follow‐up visits.^75^ Researchers recommend greater vigilance in order to recognize and acknowledge potential neurological manifestations of SARS‐CoV‐2, especially in the long‐term.^23^, ^26^

## Conclusions

In conclusion, we believe that we are seeing a pattern of parkinsonian phenotypes, reported following Covid‐19, although the available data does not yet justify a clear association between the SARS‐CoV‐2 pandemic and a potential rise in parkinsonism cases. However, since the pandemic is only two years in progression, post‐viral events can emerge at a later stage and vigilance is necessary, along with well‐defined prospective observational studies in enriched Covid‐19 infected cohorts. Such studies might also unravel the impact of vaccination. Outeiro and colleagues suggested that the underlying inflammation precipitated by SARS‐CoV‐2 might accelerate biological aging disproportionally to chronological, bringing an emergence of PD in younger age groups.^76^ With the world population longevity rising, a PD pandemic might be expected anyway, and a specific effect of Covid‐19 may be difficult to disentangle.

## Author Roles

1. Research project: A. Conception, B. Organization, C. Execution; 2. Statistical Analysis: A. Design, B. Execution, C. Review and Critique; 3. Manuscript Preparation: A. Writing of the first draft, B. Review and Critique.

IB: 1B, 1C, 3A

KRC: 1A, 1B, 3B

## Disclosures

**Ethical Compliance Statement:** We confirm that we have read the Journal's position on issues involved in ethical publication and affirm that this work is consistent with these guidelines. Informed consent was not necessary for this work. The authors confirm that the approval of an institutional review board was not required for this work.

**Funding Sources and Conflicts of Interest:** No specific funding was received for this work. The authors declare that there are no conflicts of interest relevant to this work.

**Financial Disclosures for the Previous 12 months:** The authors declare that there are no additional disclosures to report.

## References

[1] Hawkes CH , Del Tredici K , Braak H . Parkinson's disease: a dual‐hit hypothesis. Neuropathol Appl Neurobiol 2007;33(6):599–614. 17961138 10.1111/j.1365-2990.2007.00874.x PMC7194308

[2] Sulzer D . Multiple hit hypotheses for dopamine neuron loss in Parkinson's disease. Trends Neurosci 2007;30(5):244–250. 17418429 10.1016/j.tins.2007.03.009

[3] Beauchamp LC , Finkelstein DI , Bush AI , Evans AH , Barnham KJ . Parkinsonism as a third wave of the COVID‐19 pandemic? J Parkinsons Dis 2020;10(4):1343–1353. 32986683 10.3233/JPD-202211 PMC7683045

[4] Bouali‐Benazzouz R , Benazzouz A . Covid‐19 infection and parkinsonism: Is there a link? Mov Disord 2021;36(8):1737–1743. 34080714 10.1002/mds.28680 PMC8242862

[5] Brundin P , Nath A , Beckham JD . Is COVID‐19 a perfect storm for Parkinson's disease? Trends Neurosci 2020;43(12):931–933. 33158605 10.1016/j.tins.2020.10.009 PMC7577682

[6] Hoffman LA , Vilensky JA . Encephalitis lethargica: 100 years after the epidemic. Brain 2017;140(8):2246–2251. 28899018 10.1093/brain/awx177

[7] Henry J , Smeyne RJ , Jang H , Miller B , Okun MS . Parkinsonism and neurological manifestations of influenza throughout the 20th and 21st centuries. Parkinsonism Relat Disord 2010;16(9):566–571. 20650672 10.1016/j.parkreldis.2010.06.012 PMC4684089

[8] Bantle CM , Phillips AT , Smeyne RJ , Rocha SM , Olson KE , Tjalkens RB . Infection with mosquito‐borne alphavirus induces selective loss of dopaminergic neurons, neuroinflammation and widespread protein aggregation. NPJ Parkinsons Dis 2019;5:20. 31531390 10.1038/s41531-019-0090-8 PMC6744428

[9] Bopeththa B , Ralapanawa U . Post encephalitic parkinsonism following dengue viral infection. BMC Res Notes 2017;10(1):655. 29187231 10.1186/s13104-017-2954-5 PMC5708097

[10] Cubo E . Movement disorders in adult‐onset measles encephalitis. Neurologia 2003;18(1):30–33. 12590379

[11] Dehner LF , Spitz M , Pereira JS . Parkinsonism in HIV infected patients during antiretroviral therapy—data from a Brazilian tertiary hospital. Braz J Infect Dis 2016;20(5):499–501. 27449286 10.1016/j.bjid.2016.05.008 PMC9425509

[12] Guan J , Lu Z , Zhou Q . Reversible parkinsonism due to involvement of substantia nigra in Epstein‐Barr virus encephalitis. Mov Disord 2012;27(1):156–157. 21989835 10.1002/mds.23935

[13] Jang H , Boltz DA , Webster RG , Smeyne RJ . Viral parkinsonism. Biochim Biophys Acta 2009;1792(7):714–721. 18760350 10.1016/j.bbadis.2008.08.001 PMC4642437

[14] Lesteberg KE , Beckham JD . Immunology of West Nile virus infection and the role of alpha‐Synuclein as a viral restriction factor. Viral Immunol 2019;32(1):38–47. 30222521 10.1089/vim.2018.0075

[15] Marreiros R , Müller‐Schiffmann A , Trossbach SV , et al. Disruption of cellular proteostasis by H1N1 influenza a virus causes α‐synuclein aggregation. Proc Natl Acad Sci U S A 2020;117(12):6741–6751. 32152117 10.1073/pnas.1906466117 PMC7104400

[16] Pradhan S , Pandey N , Shashank S , Gupta RK , Mathur A . Parkinsonism due to predominant involvement of substantia nigra in Japanese encephalitis. Neurology 1999;53(8):1781–1786. 10563628 10.1212/wnl.53.8.1781

[17] Oliver KR , Brennan P , Fazakerley JK . Specific infection and destruction of dopaminergic neurons in the substantia nigra by Theiler's virus. J Virol 1997;71(8):6179–6182. 9223513 10.1128/jvi.71.8.6179-6182.1997 PMC191879

[18] Pasha SA , Pasha SA , Suhasini T , Rao DA . Hepatitis E virus‐associated acute encephalitic parkinsonism. J Assoc Physicians India 2018;66(3):92–93. 30341882

[19] Sadasivan S , Sharp B , Schultz‐Cherry S , Smeyne RJ . Synergistic effects of influenza and 1‐methyl‐4‐phenyl‐1,2,3,6‐tetrahydropyridine (MPTP) can be eliminated by the use of influenza therapeutics: Experimental evidence for the multi‐hit hypothesis. NPJ Parkinsons Dis. 2017;3:18. 28649618 10.1038/s41531-017-0019-z PMC5460228

[20] Tsai HH , Liou HH , Muo CH , Lee CZ , Yen RF , Kao CH . Hepatitis C virus infection as a risk factor for Parkinson disease: A nationwide cohort study. Neurology 2016;86(9):840–846. 26701382 10.1212/WNL.0000000000002307

[21] COVID‐19 Treatment Guidelines Panel. Coronavirus Disease 2019 (COVID‐19) Treatment Guidelines. National Institute of Health 2021 [cited 13/10/2021]; Available from: https://www.covid19treatmentguidelines.nih.gov/. 34003615

[22] Faber I , Brandão PRP , Menegatti F , de Carvalho Bispo DD , Maluf FB , Cardoso F . Coronavirus disease 2019 and parkinsonism: a non‐post‐encephalitic case. Mov Disord 2020;35(10):1721–1722. 32815213 10.1002/mds.28277 PMC7461093

[23] Méndez‐Guerrero A , Laespada‐García MI , Gómez‐Grande A , et al. Acute hypokinetic‐rigid syndrome following SARS‐CoV‐2 infection. Neurology 2020;95(15):e2109–e2118. 32641525 10.1212/WNL.0000000000010282

[24] Cohen ME , Eichel R , Steiner‐Birmanns B , et al. A case of probable Parkinson's disease after SARS‐CoV‐2 infection. Lancet Neurol 2020;19(10):804–805. 32949534 10.1016/S1474-4422(20)30305-7 PMC7494295

[25] Pilotto A , Masciocchi S , Volonghi I , et al. Clinical presentation and outcomes of severe acute respiratory syndrome coronavirus 2‐related encephalitis: The ENCOVID multicenter study. J Infect Dis 2021;223(1):28–37. 32986824 10.1093/infdis/jiaa609 PMC7543535

[26] Akilli NB , Yosunkaya A . Part of the Covid19 puzzle: Acute parkinsonism. Am J Emerg Med 2021;47:333.e1–e3. 33712341 10.1016/j.ajem.2021.02.050 PMC7903921

[27] Makhoul K , Jankovic J . Parkinson's disease after COVID‐19. J Neurol Sci 2021;422:117331. 33540185 10.1016/j.jns.2021.117331 PMC9755718

[28] Roy D , Song J , Awad N , Zamudio P . Treatment of unexplained coma and hypokinetic‐rigid syndrome in a patient with COVID‐19. BMJ Case Rep 2021;14(3):e239781. 10.1136/bcr-2020-239781 PMC7929831 33653852

[29] Fearon C , Mikulis DJ , Lang AE . Parkinsonism as a sequela of SARS‐CoV‐2 infection: Pure hypoxic injury or additional COVID‐19‐related response? Mov Disord 2021;36(7):1483–1484. 34043246 10.1002/mds.28656 PMC8242400

[30] Tiraboschi P , Xhani R , Zerbi SM , et al. Postinfectious neurologic complications in COVID‐19: A complex case report. J Nucl Med 2021;62(8):1171–1176. 34016729 10.2967/jnumed.120.256099 PMC8833873

[31] Rass V , Beer R , Schiefecker AJ , et al. Neurological outcome and quality of life 3 months after COVID‐19: A prospective observational cohort study. Eur J Neurol 2021;28(10):3348–3359. 33682276 10.1111/ene.14803 PMC8250725

[32] Ghosh R , Ray A , Roy D , Das S , Dubey S , Benito‐León J . Parkinsonism with akinetic mutism following osmotic demyelination syndrome in a SARS‐CoV‐2 infected elderly diabetic woman: A case report. Neurologia 2021. 10.1016/j.nrl.2021.09.007

[33] Ayele BA , Demissie H , Awraris M , et al. SARS‐COV‐2 induced parkinsonism: the first case from the sub‐Saharan Africa. Clin Park Relat Disord 2021;5:100116. 34786554 10.1016/j.prdoa.2021.100116 PMC8582125

[34] Morassi M , Palmerini F , Nici S , et al. SARS‐CoV‐2‐related encephalitis with prominent parkinsonism: clinical and FDG‐PET correlates in two patients. J Neurol 2021;21:1–8. 10.1007/s00415-021-10560-3 PMC8059684 33884450

[35] Cavallieri F , Fioravanti V , Toschi G , et al. COVID‐19 and Parkinson's disease: a casual association or a possible second hit in neurodegeneration? J Neurol 2021;269(5):1–3. 34216264 10.1007/s00415-021-10694-4 PMC8254452

[36] Ong TL , Nor KM , Yusoff Y , Sapuan S . COVID‐19 associated acute necrotizing encephalopathy presenting as parkinsonism and Myorhythmia. J Mov Disord 2022;15(1):89–92. 34781632 10.14802/jmd.21063 PMC8820880

[37] Rao AR , Hidayathullah SM , Hegde K , Adhikari P . Parkinsonism: an emerging post COVID sequelae. IDCases 2022;27:e01388. 35018281 10.1016/j.idcr.2022.e01388 PMC8733235

[38] Maeder‐Ingvar M , Prior JO , Irani SR , Rey V , Vincent A , Rossetti AO . FDG‐PET hyperactivity in basal ganglia correlating with clinical course in anti‐NDMA‐R antibodies encephalitis. J Neurol Neurosurg Psychiatry 2011;82(2):235–236. 20667855 10.1136/jnnp.2009.198697

[39] Wegner F , Wilke F , Raab P , et al. Anti‐leucine rich glioma inactivated 1 protein and anti‐N‐methyl‐D‐aspartate receptor encephalitis show distinct patterns of brain glucose metabolism in 18F‐fluoro‐2‐deoxy‐d‐glucose positron emission tomography. BMC Neurol 2014;20(14):136. 10.1186/1471-2377-14-136 PMC4076767 24950993

[40] Ellul MA , Benjamin L , Singh B , et al. Neurological associations of COVID‐19. Lancet Neurol 2020;19(9):767–783. 32622375 10.1016/S1474-4422(20)30221-0 PMC7332267

[41] Siow I , Lee KS , Zhang JJY , Saffari SE , Ng A . Encephalitis as a neurological complication of COVID‐19: A systematic review and meta‐analysis of incidence, outcomes, and predictors. Eur J Neurol 2021;28:3491–3502. 10.1111/ene.14913. 33982853 PMC8239820

[42] Guerrero JI , Barragán LA , Martínez JD , et al. Central and peripheral nervous system involvement by COVID‐19: a systematic review of the pathophysiology, clinical manifestations, neuropathology, neuroimaging, electrophysiology, and cerebrospinal fluid findings. BMC Infect Dis 2021;21(1):515. 34078305 10.1186/s12879-021-06185-6 PMC8170436

[43] Zhang H , Penninger JM , Li Y , Zhong N , Slutsky AS . Angiotensin‐converting enzyme 2 (ACE2) as a SARS‐CoV‐2 receptor: molecular mechanisms and potential therapeutic target. Intensive Care Med 2020;46(4):586–590. 32125455 10.1007/s00134-020-05985-9 PMC7079879

[44] Williams A , Branscome H , Khatkar P , et al. A comprehensive review of COVID‐19 biology, diagnostics, therapeutics, and disease impacting the central nervous system. J Neurovirol 2021;27(5):667–690. 34581996 10.1007/s13365-021-00998-6 PMC8477646

[45] Pavel A , Murray DK , Stoessl AJ . COVID‐19 and selective vulnerability to Parkinson's disease. Lancet Neurol 2020;19(9):719. 10.1016/S1474-4422(20)30269-6 PMC7434474 32822628

[46] Wan D , Du T , Hong W , et al. Neurological complications and infection mechanism of SARS‐COV‐2. Signal Transduct Target Ther 2021;6(1):406. 34815399 10.1038/s41392-021-00818-7 PMC8609271

[47] Cantuti‐Castelvetri L , Ojha R , Pedro LD , et al. Neuropilin‐1 facilitates SARS‐CoV‐2 cell entry and infectivity. Science 2020;370(6518):856–860. 33082293 10.1126/science.abd2985 PMC7857391

[48] Ulusoy A , Rusconi R , Pérez‐Revuelta BI , Musgrove RE , Helwig M , Winzen‐Reichert B , Monte DAD . Caudo‐rostral brain spreading of α‐synuclein through vagal connections. EMBO Mol Med 2013;5(7):1119–1127. 23703938 10.1002/emmm.201302475 PMC3721477

[49] Meinhardt J , Radke J , Dittmayer C , et al. Olfactory transmucosal SARS‐CoV‐2 invasion as a port of central nervous system entry in individuals with COVID‐19. Nat Neurosci 2021;24(2):168–175. 33257876 10.1038/s41593-020-00758-5

[50] Douaud G , Lee S , Alfaro‐Almagro F , et al. SARS‐CoV‐2 is associated with changes in brain structure in UK biobank. Nature 2022. 10.1038/s41586-022-04569-5 PMC9046077 35255491

[51] Matsuda K , Park CH , Sunden Y , Kimura T , Ochiai K , Kida H , Umemura T . The vagus nerve is one route of transneural invasion for intranasally inoculated influenza a virus in mice. Vet Pathol 2004;41(2):101–107. 15017022 10.1354/vp.41-2-101

[52] Mönkemüller K , Fry L , Rickes S . COVID‐19, coronavirus, SARS‐CoV‐2 and the small bowel. Rev Esp Enferm Dig 2020;112(5):383–388. 32343593 10.17235/reed.2020.7137/2020

[53] Klingelhoefer L , Reichmann H . Pathogenesis of Parkinson disease—the gut‐brain axis and environmental factors. Nat Rev Neurol 2015;11:625–636. 26503923 10.1038/nrneurol.2015.197

[54] Matschke J , Lütgehetmann M , Hagel C , et al. Neuropathology of patients with COVID‐19 in Germany: a post‐mortem case series. Lancet Neurol 2020;19(11):919–929. 33031735 10.1016/S1474-4422(20)30308-2 PMC7535629

[55] Lewis A , Frontera J , Placantonakis DG , et al. Cerebrospinal fluid in COVID‐19: A systematic review of the literature. J Neurol Sci 2021;421:117316. 33561753 10.1016/j.jns.2021.117316 PMC7833669

[56] Tufekci KU , Meuwissen R , Genc S , Genc K . Inflammation in Parkinson's disease. Adv Protein Chem Struct Biol 2012;88:69–132. 22814707 10.1016/B978-0-12-398314-5.00004-0

[57] Pissadaki EK , Bolam JP . The energy cost of action potential propagation in dopamine neurons: clues to susceptibility in Parkinson's disease. Front Comput Neurosci 2013;7:13. 23515615 10.3389/fncom.2013.00013 PMC3600574

[58] Dale RC , Church AJ , Surtees RA , et al. Encephalitis lethargica syndrome: 20 new cases and evidence of basal ganglia autoimmunity. Brain 2004;127(Pt 1):21–33. 14570817 10.1093/brain/awh008

[59] Haider A , Siddiqa A , Ali N , Dhallu M . COVID‐19 and the brain: acute encephalitis as a clinical manifestation. Cureus 2020;12(10):e10784. 33154851 10.7759/cureus.10784 PMC7609129

[60] Huo L , Xu KL , Wang H . Clinical features of SARS‐CoV‐2‐associated encephalitis and meningitis amid COVID‐19 pandemic. World J Clin Cases 2021;9(5):1058–1078. 33644169 10.12998/wjcc.v9.i5.1058 PMC7896657

[61] Wu X , Wu W , Pan W , Wu L , Liu K , Zhang H‐L . Acute necrotizing encephalopathy: an underrecognized clinicoradiologic disorder. Mediators Inflamm 2015;2015:792578. 25873770 10.1155/2015/792578 PMC4385702

[62] Baghdadi Y , Anh SJ , Brook A , Moadel R , Freeman LM . Unilateral absence of the basal ganglia on 123I‐Ioflupane DaTScan. Clin Nucl Med 2019;44(10):842–843. 31348084 10.1097/RLU.0000000000002743

[63] García C , de León S , Cabello JP , Ortiz R , Vaamonde J . Parkinsonism associated with pathological 123I‐FP‐CIT SPECT (DaTSCAN) results as the initial manifestation of sporadic Creutzfeldt‐Jakob disease. Case Rep Neurol Med 2018;2018:5157275. 29955403 10.1155/2018/5157275 PMC6000879

[64] de Souza A . Movement disorders and the osmotic demyelination syndrome. Parkinsonism Relat Disord 2013;19(8):709–716. 23660544 10.1016/j.parkreldis.2013.04.005

[65] Siddiqi HK , Libby P , Ridker PM . COVID‐19—A vascular disease. Trends Cardiovasc Med 2021;31(1):1–5. 33068723 10.1016/j.tcm.2020.10.005 PMC7556303

[66] Tanner CM , Ottman R , Goldman SM , et al. Parkinson disease in twins: an etiologic study. JAMA 1999;281(4):341–346. 9929087 10.1001/jama.281.4.341

[67] Meng L , Shen L , Ji HF . Impact of infection on risk of Parkinson's disease: a quantitative assessment of case‐control and cohort studies. J Neurovirol 2019;25(2):221–228. 30632012 10.1007/s13365-018-0707-4

[68] Fang F , Wirdefeldt K , Jacks A , Kamel F , Ye W , Chen H . CNS infections, sepsis and risk of Parkinson's disease. Int J Epidemiol 2012;41(4):1042–1049. 22523201 10.1093/ije/dys052 PMC3429872

[69] Tsui JK , Calne DB , Wang Y , Schulzer M , Marion SA . Occupational risk factors in Parkinson's disease. Can J Public Health 1999;90(5):334–337. 10570579 10.1007/BF03404523 PMC6979620

[70] Merello M , Bhatia KP , Obeso JA . SARS‐CoV‐2 and the risk of Parkinson's disease: facts and fantasy. Lancet Neurol 2021;20(2):94–95. 33253627 10.1016/S1474-4422(20)30442-7 PMC7834123

[71] Nirenberg MJ . New‐onset movement disorders in COVID‐19: Much ado about nothing? Tremor Other Hyperkinet Mov (N Y) 2021;11:31. 34395056 10.5334/tohm.644 PMC8323522

[72] Neumann B , Schmidbauer ML , Dimitriadis K , et al. Cerebrospinal fluid findings in COVID‐19 patients with neurological symptoms. J Neurol Sci 2020;418:117090. 32805440 10.1016/j.jns.2020.117090 PMC7417278

[73] Worldmeter.info. COVID‐19 CORONAVIRUS PANDEMIC. Dover, Delaware, U.S.A. 2021 [updated 24/10/2021]; Available from: https://www.worldometers.info/coronavirus/.

[74] Tysnes OB , Storstein A . Epidemiology of Parkinson's disease. J Neural Transm (Vienna) 2017;124(8):901–905. 28150045 10.1007/s00702-017-1686-y

[75] COVID‐19 Clinical Neuroscience Study (COVID‐CNS) . 2021, Available from: https://www.liverpool.ac.uk/covid-clinical-neuroscience-study/.

[76] Outeiro TF , Krisko A . Reply to: “Parkinson's disease and COVID‐19: do we need to be more patient?”. Mov Disord 2021;36(2):278–279. 33599004 10.1002/mds.28482 PMC8013411

